# Who is more trustworthy? The influence mechanism of AI vs. human doctor triage on user trust: testing the mediating effect of psychological distance, and a multiple moderating effects analysis

**DOI:** 10.3389/fpsyg.2025.1730902

**Published:** 2026-01-12

**Authors:** Jinghao Chen, Huayang Wang, Xiaoyu Qiu

**Affiliations:** 1 School of Public Policy and Management, Guangxi University, Nanning, China; 2 Regional Social Governance Innovation Research Center, Guangxi University, Nanning, China

**Keywords:** AI technology adoption level, anthropomorphism, medical triage, psychological distance, task sensitivity, user trust

## Abstract

**Purpose/significance:**

To optimize smart healthcare services and advance the sustainable deployment of AI in medical triage, this study investigates differences in user trust between AI and human medical triage doctors and the underlying psychological mechanisms.

**Methods/procedures:**

Four online experiments were conducted using a between-group design to systematically manipulate the medical triage doctors (AI vs. human), degree of anthropomorphism (high vs. low), task sensitivity (high vs. low), and AI technology adoption level (high vs. low). Participants were recruited online to view medical triage engagement screenshots, and respond to measures assessing perceived psychological distance, anthropomorphism, task sensitivity, AI technology adoption level, and user trust. Process macros were used to test the mediation and moderation effects.

**Results/conclusions:**

The study found that (1) participants placed greater trust in human than in AI medical triage doctors; (2) psychological distance played a partial mediating role; (3) a high degree of anthropomorphism effectively reduced the psychological distance between participants and AI medical triage doctors; (4) in low task sensitivity scenarios, there was no significant difference in psychological distance from high-anthropomorphism AI and human medical triage doctors, and both were perceived as closer than low-anthropomorphism AI medical triage doctors. In high task sensitivity scenarios, psychological distance was closest for human medical triage doctors, followed by high-anthropomorphism AI medical triage doctors, and farthest for low-anthropomorphism AI medical triage doctors; and (5) high AI technology adoption level diminished the trust disparity between AI and human medical triage doctors; however, participants still exhibited a higher level of trust in human medical triage doctors. These results emphasize the importance of considering psychological distance in AI healthcare trust research, revealing the task reliance of anthropomorphism. The study also develops a comprehensive trust model that incorporates various moderating influences.

## 1 Introduction

A new wave of technological innovation, led by Artificial Intelligence (AI), is reshaping today’s healthcare practices. In particular, large language models (LLMs) such as ChatGPT are catalyzing rapid advances in AI healthcare (Chen et al., 2024; Lu et al., 2023). AI-utilization is now present throughout the entire healthcare continuum, from smart hospital administration and drug-targeting identification to early disease screening (Jiang et al., 2017; Yu et al., 2018; Keskinbora, 2019; Attia et al., 2019), operating with unmatched breadth and depth. The profound integration of AI and healthcare has not only accelerated the progression of medical technology, it has also emerged as a central concern in general society. With regards to human-AI collaboration, calibrating users’ reliance so that they neither over-rely on nor categorically distrust AI systems, but instead achieve appropriate reliance, has become a central topic in human factors and automation-trust research (Lee and See, 2004), offering an important lens through which to understand trust in AI systems in healthcare.

Increasing demand for healthcare services presents significant problems to the traditional “patient-to-department” arrangement. Patients frequently register for inappropriate departments due to a lack of medical knowledge, or suffer long wait times due to misunderstanding hospital protocols or departmental practitioners’ lack of specialist knowledge, leading to ineffective care and unsatisfactory experiences. Medical personnel, overwhelmed by heightened service demands, find it challenging to deliver individualized treatment, leading to intensified doctor-patient conflicts (Cui and Liu, 2020; Tyler et al., 2024). To address these issues, National Health Commission of the People’s Republic of China released a policy guideline in November 2024 titled Reference Guidelines for Artificial Intelligence Application Scenarios in the Health Care Industry, which explicitly designated “AI + triage” as a priority area of development (National Health Commission of the People’s Republic of China, 2024). As a result, AI medical triage doctors have been increasingly proposed and developed. (Jiang et al., 2017). Through close integration of AI with clinical workflows, doctors are able to utilize big data, natural language processing, and deep learning to automate the collection of patient histories, generate preliminary diagnostic suggestions, and direct patients to appropriate care pathways, thereby easing clinicians’ workload (Tyler et al., 2024; Steerling et al., 2025). This has enabled shifts from “patients seeking departments” to “departments seeking patients,” and from “blind registration” to “precision services,” to ultimately deliver a far more streamlined, patient-centered care experience (Leachman and Merlino, 2017). Nevertheless, despite the significant benefits AI medical triage doctors can provide, their functions as primary healthcare doctors continue to be under public scrutiny and judgment. Studies suggest that the intricate and multifaceted characteristics of medical situations as well as the “black-box” nature and uncertainty of AI algorithms lead to widespread skepticism among users regarding AI medical triage doctors, affecting its advancement and implementation within healthcare practices (Cui and Liu, 2020; Hamet and Tremblay, 2017). Research suggests that integrating Theory of Mind (ToM) and Metacognition (MC) into AI processes can significantly enhance the social and self-regulatory capabilities of intelligent systems. Specifically, when interacting with humans and other agents, ToM improves the social cognition and interaction quality of AI, while MC helps the AI monitor and correct its own behavior, thereby reducing errors and increasing autonomy (Bamicha and Drigas, 2024). However, even when AI medical triage doctors perform as well as or better than human medical triage doctors on objective diagnostic tasks, patients may still reject medical decisions made by AI algorithms because of concerns about moral responsibility and a perceived lack of warmth or human touch, a phenomenon that has been described as “resistance to medical AI” (Longoni et al., 2019). Patients’ attitudes toward AI medical triage doctors are therefore not determined solely by accuracy or convenience, but are also strongly shaped by affective experience and ethical concerns.

Despite its technical power, then, AI integration in healthcare should aim to be a cognitive amplifier and assistive tool for medical professionals rather than as a full replacement for human medical triage doctors. In populations with neurodevelopmental disorders and other complex conditions, for example, AI tools can provide valuable support in assessment and communication, but they should remain under the supervision of human medical triage doctors’ professional judgment (Moraiti and Drigas, 2023). Furthermore, in the development and deployment of medical AI, it is crucial to emphasize adherence to ethical principles concerning data protection, safeguards against algorithmic bias, transparency and explainability, and clear accountability mechanisms, prioritizing the assistive and augmentative uses of AI rather than its replacement possibilities. Such practices are key to fostering more positive user attitudes and understandings regarding the use of intelligent systems in healthcare (Solanki et al., 2023; Palaniappan et al., 2024).

Consequently, it is of considerable practical significance to explore the trust disparity between AI and human medical triage doctors, along with its contributing elements, for the advancement of AI technology adoption within medical triage practices, and to enable the sustainable development of AI medical triage doctors. Therefore, this study concentrates on the following fundamental inquiries:

(1) In medical triage services, can users’ trust in AI medical triage doctors equal their trust in traditional human medical triage doctors? In other words, who do patients find to be more trustworthy: AI or human medical triage doctors?
(2) What impact do medical triage doctors have on user trust, and what psychological mechanisms might explain trust disparities?
(3) Are there particular task-related environmental elements or individual user traits that can influence the establishment of this trust relationship?

## 2 Literature review

AI adoption in medicine involves the use of complex algorithms and methods, such as machine learning, to analyze medical data and support clinical decision-making. By thoroughly analyzing vast amounts of medical data, including imaging and electronic health records, AI seeks to replicate the cognitive capabilities of human medical triage doctors to provide cognitive aids and decision-making support for various tasks, including disease diagnosis, treatment planning, and patient health management (Rajpurkar et al., 2022; Moor et al., 2023). Due to advances in digital health technologies, AI is no longer confined to analyzing static medical records. By integrating electronic health records, medical imaging, wearable sensors, and remote monitoring data, AI extends clinical assessment from a single encounter to continuous monitoring, helping doctors identify risks earlier and more accurately, and allowing for the development of individualized treatment plans. In this process, machine learning methods such as neural networks, support vector machines, decision trees, and rule-based classifiers are widely used to build disease prediction and classification models (Bamicha et al., 2024; Bamicha et al., 2025), substantially improving the speed and accuracy of diagnosis and clinical decision-making.

Scholars worldwide have been exploring the driving role of big data in healthcare, conducting extensive work on core areas such as multimodal data fusion and knowledge graph construction. This body of research consistently shows that AI holds substantial promises for basic medical research as well as disease diagnosis and treatment; on certain well-defined tasks, its diagnostic accuracy and efficiency have even surpassed those of human medical triage doctors (Zhu et al., 2024; Contreras and Vehi, 2018; Haenssle et al., 2018; MacIntyre et al., 2023; Ting et al., 2019). For example, AlphaFold successfully predicted the three-dimensional structures of proteins using deep learning (Jumper et al., 2021). In diagnostics, AI medical triage doctors have demonstrated the ability to identify lung nodules, breast cancer (Contreras and Vehi, 2018), and various skin diseases (Haenssle et al., 2018). Furthermore, IBM’s Watson not only aids in diagnosing heart conditions (Hutson, 2017), but has also shown higher accuracy than human medical triage doctors in over a thousand cancer diagnoses (Lohr, 2016). Driven by this strong technological foundation, AI medical triage is rapidly emerging as a key application of AI in the healthcare service process. Initially, AI medical triage doctors functioned primarily as a public-facing, self-service tool for consultation and triage. Based predominantly on rules and machine learning, its goal was to provide patients with triage recommendations on whether to seek medical attention, and where to do so (Baker et al., 2020). Later, AI medical triage doctors manifested as physical robots providing initial consultations, appointment scheduling, information retrieval, and hospital navigation services, effectively mitigating conventional healthcare challenges such as appointment booking difficulties, appointment inaccuracies, and prolonged wait times. With the widespread adoption of mobile internet, triage services swiftly transitioned to online platforms, such as official accounts and mini-programs. Applications like Tencent Miying and Babylon Health transcended temporal and spatial limitations, allowing patients to participate in remote initial consultations and pre-consultation services at any time, from any location. By processing textual or vocal descriptions from patients, intelligent AI medical triage doctors employed backend algorithms to deliver accurate triage advice as well as offering relevant disease-related information. Subsequently, AI medical triage doctors progressed into intelligent wearable devices, transitioning from the passive “question-and-answer” format to proactive “health management” (Dias and Paulo, 2018; Xie et al., 2021). Current AI medical triage doctors have evolved to an advanced stage, characterized by the integration of cutting-edge technologies such as natural language processing and medical LLMs which allow for in-depth analysis of multi-source, heterogeneous data across the entire clinical workflow. Unlike traditional question-answer systems which were reliant on fixed rules or knowledge graphs, the latest generation of AI medical triage doctors, driven by LLMs, are able to handle complex, open-ended questions with superior flexibility, generating logical and coherent in-depth responses, ultimately redefining the quality and dynamics of patient–provider interactions (Xu et al., 2024; Shaheen, 2021). In summary, AI medical triage doctors have transformed from a singular technological application into a holistic, multi-modal intelligent solution encompassing the full healthcare continuum.

Despite notable progress in the technology, applications, and functions of AI medical triage doctors, several gaps remain. First, existing research lacks a systematic account of trust mechanisms. Most studies have focused on diagnostic accuracy, efficiency, and system performance, or on the general user acceptance of AI in healthcare (Baker et al., 2020; Ilicki, 2022; Townsend et al., 2023; Tyler et al., 2024), rarely exploring users’ deeper psychological perceptions – especially that of psychological distance – to unpack trust differences between AI and human medical triage doctors and the mechanisms underlying them. Second, research contexts and variables have generally been constrained. Numerous studies have been limited to individual variables or particular situations, neglecting to thoroughly investigate the interconnectivity among various components. Much of the existing research has focused on macro, conceptual level and lacked systematic empirical testing in concrete medical triage tasks and settings of how multiple factors might shape users’ trust in and reliance on AI. Third, inadequate consideration has been given to the individual patient differences. Current studies have rarely explored how individual user characteristics influence their trust in AI medical triage doctors. Debates on the normative use of AI in healthcare from ethical or governance perspectives have increasingly emphasized the need for actionable developer guidelines and regulatory frameworks addressing data protection, safeguards against algorithmic bias, transparency and explainability, and clear accountability mechanisms (Solanki et al., 2023; Palaniappan et al., 2024). However, relatively little is known about how the individual characteristics of users might moderate their trust in AI medical triage.

Therefore, the current study incorporates psychological distance in exploring the effectiveness of AI medical triage doctors, allowing for the development of a comprehensive model that includes both mediating and multiple moderating effects to explain gaps. This study seeks to thoroughly investigate the fundamental question of whether patients find AI or human medical triage doctors to be more reliable, offering focused theoretical and empirical insights for the enhancement and application of AI medical triage doctors going forward.

## 3 Study hypotheses

### 3.1 The effect of medical triage doctors (AI vs. human) on user trust

The adoption of medical services hinges critically on the trust of the patient. The Competence–Personality Theory posits that individuals prioritize two essential needs when selecting between human and AI decision-making: functional needs (i.e., objective criteria for effective task execution) and psychological needs (i.e., subjective aspirations for recognition as distinct individuals; Qin et al., 2025). In comparison to conventional healthcare models, AI medical triage doctors provide enhanced, more convenient, and more efficient medical services (Shukur et al., 2024). Nonetheless, these AI medical triage doctors are devoid of emotion, empathy, and human warmth, hindering their ability to deliver individualized guidance with a human touch. Their decision-making processes are characterized by a deficiency in transparency and interpretability, and they are incapable of delivering appropriate psychological support or communication akin to that of human medical triage doctors (Shukur et al., 2024; Harada et al., 2020; Chan, 2023). Numerous studies have demonstrated that patients prefer healthcare services performed by human medical triage doctors (Longoni et al., 2019). Human medical triage doctors are able to render more empathetic and adaptable decisions informed by experience, intuition, ethical considerations, and patient requirements (Shukur et al., 2024). Even when AI medical triage doctors responses achieve or exceed the accuracy of human medical triage doctors, AI medical triage doctors remain fundamentally auxiliary tools under the supervision of human medical triage doctors (Von Eschenbach, 2021) and act as a complement to, rather than a replacement of, human medical triage doctors (Moraiti and Drigas, 2023). Patients routinely exhibit heightened trust in human medical triage doctors (Riedl et al., 2024). Consequently, the following hypothesis was proposed:

*Hypothesis 1 (H1)*: In contrast to the AI medical triage doctor, the human medical triage doctor is more likely to engender user trust.

### 3.2 The mediating effect of psychological distance

Psychological distance denotes the subjective experience of proximity regarding time, space, and other dimensions that humans associate with people, events, and things in their cognition, emotions, and behavior (Trope and Liberman, 2010). The Theory of Interpretive Levels (Liberman and Trope, 2008) posits that subjective perceived distance significantly impacts cognitive information processing, as well as individuals’ judgments, evaluations, predictions, choices (Park and Park, 2016; Trope and Liberman, 2003), and behaviors (Trope and Liberman, 2010). Comprehensive research findings indicate a strong correlation between psychological distance and trust, whereby diminishing psychological distance markedly increases trust (Wang et al., 2008; Bandara et al., 2021; Park et al., 2024).

Human medical triage doctors, having intrinsically human attributes such as social presence, empathy, and emotional resonance, are better equipped to bridge the psychological gap between them and their patients (Ahn et al., 2021; Chin et al., 2024). Conversely, AI medical triage doctors have been shown to demonstrate increased psychological detachment from patients owing to constraints in their emotional empathy and intricate communication abilities (Hsieh and Lee, 2024; Xiong et al., 2024). Therefore, the following hypothesis was proposed:

*Hypothesis 2 (H2)*: Psychological distance plays a positive mediating role in the influence of medical triage doctors (both AI and human) on user trust.

### 3.3 The moderating effect of anthropomorphism

Anthropomorphism is the assignment of human-like traits, motives, intents, or feelings to actual or fictional non-human entities (Epley et al., 2007; Epley et al., 2008). Current studies have demonstrated that anthropomorphism designs, such as human-like figures or mascots, can markedly diminish psychological distance between users and non-human intelligent agents (Ali et al., 2021; Chung and Han, 2022; Guido and Peluso, 2015; Li and Sung, 2021). In 1970, Japanese robotics expert Masahiro Mori introduced the Uncanny Valley effect (Mori et al., 2012), which posits that within a specific range, human attraction toward robots escalates with the increase in their anthropomorphism characteristics, but when an entity becomes almost human yet still imperfect, people may experience feelings of eeriness, discomfort, or dislike, causing affinity (and potentially trust) to drop sharply—forming the “valley” (Mori et al., 2012). When AI attains a specific degree of anthropomorphism, before triggering the Uncanny Valley effect, its proficiency at mimicking human interaction, offering emotional support, and exhibiting adaptive cognition is markedly enhanced (Waytz et al., 2010). At lower levels of anthropomorphism, the non-human characteristics of AI become increasingly apparent, prompting users to favor trust and rapport with actual human medical triage doctors. Provided anthropomorphism remains below the uncanny-valley threshold, the ability of AI to mimic human dialogue and provide affective support helps bridge the psychological distance between it and its users, and through this aligned perceived proximity, its human-like interface can replace certain functions of physicians (Yang et al., 2023). Therefore, the following hypotheses were proposed:

*Hypothesis 3 (H3)*: The degree of anthropomorphism influences the effect of the AI medical triage doctor on users’ perceived psychological distance.

*Hypothesis H3a (H3a)*: When anthropomorphism of AI is minimal, the human medical triage doctor is able to establish a closer psychological distance with users than the AI medical triage doctor.

*Hypothesis 3b (H3b)*: When the degree anthropomorphism is high, no substantial disparity is seen in users’ perceived psychological distance with AI and human medical triage doctors.

### 3.4 The remodulating effect of task sensitivity

Task sensitivity denotes the extent to which individuals recognize privacy hazards, societal pressures, and possible adverse outcomes while discussing particular health concerns (Liang et al., 2018). Privacy Computing Theory posits that internet or computer users evaluate potential advantages against potential dangers prior to revealing personal information. As perceived threats escalate, users’ psychological demand for security and trust intensifies (Kim et al., 2019; Wang et al., 2024).

In low task sensitivity scenarios (e.g., consultations for common, non-sensitive illnesses), users perceive lower risks in revealing information. In such scenarios, both human and high-anthropomorphism AI medical triage doctors are capable of delivering human-like encounters are able to cultivate a sense of psychological closeness in the user. In contrast, low-anthropomorphism AI medical triage doctors, which are devoid of human traits, foster increased psychological distance from users. In high task sensitivity scenarios (e.g., consultations involving extremely sensitive or private issues or severe health conditions), users’ expectations of their service-providing doctor’s professionalism, empathy, and privacy safeguards increase significantly. Currently, human medical triage doctors utilize their distinctive interpersonal warmth and intuitive talents to foster a sense of security and trust that is difficult for AI medical triage doctors to emulate, allowing the human doctors to create a closer psychological connection with the patient. Even a high-anthropomorphism AI medical triage doctor may evoke user apprehension due to its non-human characteristics, increasing the psychological distance experienced by the patient, compared to the psychological distance they perceive with a human medical triage doctor. The robotic characteristics of low-anthropomorphism AI exacerbates this disadvantage, resulting in even more psychological distance. As such, the following hypothesis was proposed:

*Hypothesis 4 (H4)*: The moderating effect of anthropomorphism is further mitigated by task sensitivity.

*Hypothesis 4a (H4a)*: In low task sensitivity scenarios, users experience no significant difference in perceived psychological distance from either the human or the high-anthropomorphism AI medical triage doctors. Furthermore, users’ perceived psychological distance from both doctors is much less than that with the low-anthropomorphism AI medical triage doctor.

*Hypothesis 4b (H4b)*: In high task sensitivity scenarios, the human medical triage doctor will be regarded as having the closest psychological distance to users, followed by the high-anthropomorphism AI medical triage doctor, while users perceive the furthest psychological distance from the low-anthropomorphism AI medical triage doctor.

### 3.5 The moderating effect of users’ level of AI adoption

AI technology adoption level refers to users’ approval of both the functional and emotional dimensions of AI – affecting their willingness to utilize AI tools – which are fundamentally rooted in one’s pre-existing attitudes and subjective views of new technologies. This concept has been examined in-depth through the Technology Acceptance Model (TAM; Na et al., 2022) as well as the Unified Theory of Acceptance and Use of Technology (UTAUT; Na et al., 2023). In the context of AI technology’s growing incorporation into healthcare services, users’ previous opinions and acceptance levels toward AI technology may influence their trust in both AI and human medical triage doctors.

Individuals exhibiting an elevated level of adoption of AI technology demonstrate increased receptiveness toward novel technologies, enhanced awareness of AI’s potential in healthcare, and greater understanding of the benefits of AI in improving efficiency, objectivity, and continuous service (Esfandiari et al., 2024). As a result, individuals with a high adoption level of AI may be more inclined to trust AI medical triage doctors, and may regard AI medical triage doctors to be comparable to human medical triage doctors in specific respects. Conversely, people with a lower level of AI adoption are more prone to exhibit heightened suspicion and resistance toward the reliability of AI medical triage doctors, as well as of claims of them demonstrating an ability for “human touch” (Chen et al., 2022). Overall, users’ confidence in human medical triage doctors markedly surpasses their confidence in AI medical triage doctors. Therefore, the following hypotheses was proposed:

*Hypothesis 5 (H5)*: A user’s level of adoption of AI technology moderates the impact of the medical triage doctor on user trust.

*Hypothesis 5a (H5a)*: Among users with a high level of AI technology adoption, no substantial disparity exists between the AI and human medical triage doctors in terms of user trust.

*Hypothesis 5b (H5b)*: Among users who have a low level of adoption of AI technology, the human medical triage doctor engenders increased trust in users than the AI medical triage doctors.

**Figure 1:**
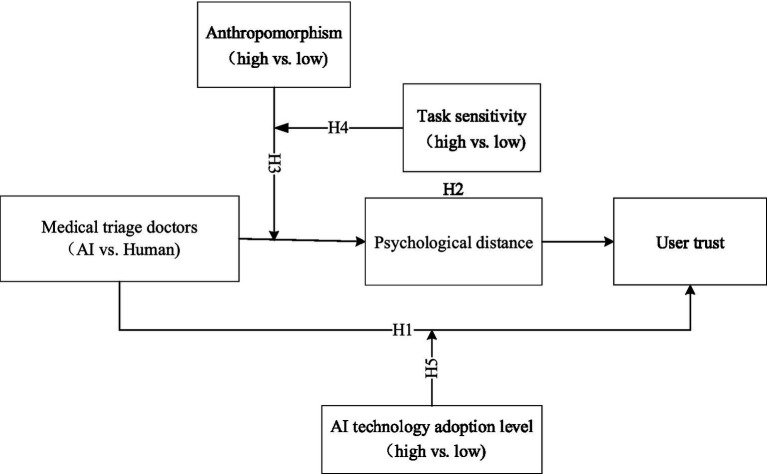
Research framework. *** *p* < 0.001.

The study framework is presented in Figure 1.

## 4 Study design

### 4.1 Experimental design

This study examined the influence of medical triage doctors (AI vs. human) on user trust and the mechanisms involved, utilizing four separate experiments. Study 1 employed a single-factor, two-level (medical triage doctor: AI vs. human) between-subjects experimental design to investigate the effect of the doctor on user trust and the mediating effect of psychological distance. Study 2 employed a 2 (medical triage doctor: AI vs. human) × 2 (anthropomorphism: high vs. low) between-subjects design. This experiment incorporated anthropomorphism as a moderator to assess its impact on the relationship between the medical triage doctor and user psychological distance. Study 3 employed a 2 (medical triage doctor: AI vs. human) × 2 (anthropomorphism: high vs. low) × 2 (task sensitivity: high vs. low) between-subjects experimental framework. Task sensitivity was incorporated as an additional moderating variable to investigate the interplay between the medical triage doctors and the degree of anthropomorphism attributed to the AI medical triage doctor across various task sensitivity conditions. Study 4 employed a 2 (medical triage doctor: AI vs. human) × 2 (AI technology acceptance: high vs. low) between-subjects experimental design to investigate the moderating effect of users’ AI technology acceptance on the impact of the triage medical doctor on user trust.

### 4.2 Manipulation design of medical triage doctors, anthropomorphism, and task sensitivity

To help participants accurately distinguish between the two types of medical triage doctors, the manipulation of doctor identity was based on the research conducted by Wang et al. (2021). In Study 1, the human medical triage doctor was depicted by a still image of an actual human doctor (with a neutral facial expression), supplemented by the doctor’s name and textual introductions written in the first person. The AI medical triage doctor was depicted by a robotic-looking customer service avatar. With the exception of the avatar image and name, all elements of the human and AI medical triage doctors were the same (see Appendix 1 for details). “Headache” was chosen as the experimental scenario for two reasons. First, as a high-frequency complaint presented in frontline consultations, it was similar to everyday experiences, which would enable participants to empathize with the scenario and immerse themselves more quickly, thus reducing comprehension burden and social-desirability bias (Minen et al., 2020). Second, the symptom satisfies the ethical requirement of minimal risk and, under controlled conditions, is well-suited to examining how differences in triage providers elicit changes in trust and psychological distance (Lin et al., 2021).

In Study 2, the introductory interface for the high-anthropomorphism AI medical triage doctor was the same used for the human medical triage doctor in Study 1, except that the AI medical triage doctor in Study 2 was explicitly identified as such. The introductory interface for the low-anthropomorphism AI medical triage doctor was presented visually with a human-like face, but no first-person pronouns nor names were used in the written introduction (see Appendix 2 for details).

In Study 3, the medical triage doctor presentation was the same as in Study 2, but the interaction content differed based on low vs. high task sensitivity. The modulation of high and low task sensitivity in Study 3 was formulated by adapting and developing content following the classifications of Pescosolido (2015) and Kokolakis (2017). Four medical triage task scenarios with low task sensitivity were developed, as well as four with high task sensitivity. A sample of 31 participants (13 males) participated in a straightforward voting process to identify the most representative scenarios, which were also considered to be the most appropriate scenarios to use for each level. The low task sensitivity scenario which garnered the largest number of votes (17 votes, 54.84%) was as follows: A patient presents with a sore throat, slight cough, and nasal congestion, seeking a diagnosis and self-care recommendations. The high task sensitivity scenario which garnered the garnered the most votes (18 votes, 58.06%) was as follows: A patient presents with a month-long low-grade fever, nausea, vomiting, substantial weight loss (approximately 10 pounds), recurrent sore throat, and swollen lymph nodes, raising suspicion of a human immunodeficiency virus (HIV) infection.

In Study 4, the manipulations for the medical triage doctor and the interaction content were identical to those in Study 1 (see Appendix 3 for details).

### 4.3 Questionnaire design and pre-experimentation

The scales used in this study were adapted from established scales published in various international journals, modified appropriately to fit the research context while ensuring content validity. The specific measurement items are presented in Table 1.

**Table 1:** Variable measurement scales.

Measured variable	Measurement Items	References
Visual presentation of medical triage doctor	To what extent do you believe that this doctor is a human medical triage doctor?	(Youn and Jin, 2021)
	To what extent do you believe that this doctor is an AI medical triage doctor?	
Psychological distance	I feel very close to this medical triage doctor during our interaction.	(Li and Sung, 2021; Chen and Li, 2018)
	I feel no sense of distance when interacting with this medical triage doctor.	
	My interaction with this medical triage doctor is smooth and comfortable.	
	This medical triage doctor is able to understand my true needs and feelings.	
	This medical triage doctor can empathize with my situation.	
Anthropomorphism	This medical triage doctor looks like an actual human medical triage doctor.	(Bartneck et al., 2009; Waytz et al., 2010; Wang et al., 2021)
	This medical triage doctor seems to have their own thoughts and judgments.	
	This medical triage doctor seems able to understand my needs and responds accordingly.	
	This medical triage doctor seems able to understand my mood and feelings.	
	This medical triage doctor seems to genuinely want to help me solve my problem.	
Task sensitivity	This type of medical scenario is sensitive and not suitable for public discussion.	(Smith et al., 1996; Malhotra et al., 2004)
	This type of medical scenario is generally considered to be personal and private.	
	Consulting about this type of medical scenario makes me feel anxious or worried.	
	I worry that others will have a negative opinion of me for consulting about this type of medical scenario.	
AI technology adoption level	I believe that AI technology will improve my life.	(Davis, 1989; Grassini, 2023)
	I believe that AI technology can improve my learning and work efficiency.	
	I believe that AI technology is reliable and safe.	
	I believe that AI technology is beneficial to humanity.	
	I believe that I will use AI technology in the future.	
User trust	This medical triage doctor is honest.	(Everard and Galletta, 2005; Lee and Choi, 2017)
	This medical triage doctor is trustworthy.	
	This medical triage doctor has the professional knowledge and ability to solve my problem.	
	The information, services, and advice provided by this medical triage doctor are credible.	

The independent variable, medical triage doctors, was assessed using a two-item scale derived from Youn and Jin (2021). The two items evaluated the manipulation check for the doctor type: “To what extent do you believe that this doctor is a human medical triage doctor?” and “To what extent do you believe that this doctor is an AI medical triage doctor?” Both items were rated using a seven-point Likert scale (1 = strongly disagree, 7 = strongly agree).

The mediating variable of psychological distance was assessed using a five-item scale derived from the research of Li and Sung (2021) and Chen and Li (2018). An example item is: “I feel very close to this medical triage doctor during our interaction.” Higher scores on this scale signified a diminished perceived psychological gap between the user and the medical triage doctor.

The moderating variable, anthropomorphism, was assessed using a five-item scale developed from the research of Bartneck et al. (2009), Waytz et al. (2010), and Wang et al. (2021). An example item is: “This medical triage doctor looks like an actual human medical triage doctor.” Higher scores indicated an increased level of perceived anthropomorphism. The task sensitivity scale had four items, and was based on the studies conducted by Smith et al. (1996) and Malhotra et al. (2004). An example item is: “This type of medical scenario is sensitive and not suitable for public discussion.” Higher scores signified an increased degree of task sensitivity. The four-item AI technology adoption level scale was developed based on the research of Davis (1989) and Grassini (2023). An example item is: “I believe that AI technology will improve my life.” Higher ratings signified an increased degree of user adoption of AI technology.

The dependent variable, user trust, was assessed using a four-item scale derived from the research of Everard and Galletta (2005) and Lee and Choi (2017). An example item is: “This medical triage doctor is honest.” Higher scores on the scale signified an increased level of user trust in the medical triage doctor.

Several measures were also incorporated in the study as control variables and manipulation assessments. Participants’ previous acceptance of new technology items was assessed using the question, “How would you rate your acceptance of innovative technology products (e.g., AI, smart devices)?” The item was rated using a seven-point scale (1 = extremely reluctant, 7 = extremely willing to try new technologies). Participants’ prior level of trust in AI medical products was assessed using the question, “Do you already trust a range of AI medical products or services, such as AI medical triage doctors?” The item was rated using a seven-point scale (1 = highly distrustful, 7 = strongly trusting). Scenario participation was assessed using a single item derived from Wang et al. (2021): “Can you envision yourself as the patient in the scenario?” (1 = not at all, 7 = completely). This strategy aimed to mitigate potential interference in the results due to insufficient involvement with the experimental circumstances.

Before the formal experiments began, three pre-experiments were executed. The objective of the initial pre-examination was to ascertain whether participants could accurately differentiate between the two medical triage doctors manipulated in Study 1. We recruited 49 participants (25 males) through the Credamo survey platform. Participants were randomly assigned to one of two groups, and presented with the image and introduction of the medical triage doctor. They were then asked to evaluate the doctor after viewing the full presentation. An independent samples *t*-test indicated that, under the human medical triage doctor condition, participants’ evaluation of the AI medical triage doctor was considerably lower than that of the human medical triage doctor (M_Human medical triage doctor_ = 4.83, SD = 0.92 vs. M_AI medical triage doctor_ = 2.96, SD = 1.21; *t* = −6.099, *p* < 0.001). The elevated recognition score demonstrated that the participants distinctly identified the target as being a human medical triage doctor. Similarly, in the AI medical triage doctor condition, the rating for the AI medical triage doctor was significantly higher than the rating for the human medical triage doctor (M_AI medical triage doctor_ = 5.08, SD = 1.26 vs. M_Human medical triage doctor_ = 3.54, SD = 1.47; *t* = 3.926, *p* < 0.001), indicating that the participants correctly identified the medical triage doctor as being AI. Thus, the experimental materials satisfactorily fulfilled the manipulation check criteria, thereby supporting the commencement of the official experiment.

The objective of the second pre-experiment was to ascertain participants’ ability to accurately differentiate between the high- and low-anthropomorphism AI medical triage doctors. The methodology followed that used in Study 1. A total of 73 participants (37 males) assessed the level of the medical triage doctor’s anthropomorphism (*α* = 0.916). An independent samples *t*-test indicated that the high-anthropomorphism AI medical triage doctor received a substantially higher rating for anthropomorphism as compared to the low-anthropomorphism AI medical triage doctor (M _High-anthropomorphism_ = 5.35, SD = 1.08 vs. M _Low-anthropomorphism_ = 4.66, SD = 1.41; *t* (71) = 2.356, *p* < 0.05). This validated the effective manipulation of the AI medical triage doctors’ level of anthropomorphism.

The objective of the third pre-experiment was to confirm the effective modulation of task sensitivity. Eighty-five participants (47 males) were randomly allocated to either a high or low task-sensitivity scenario, and were asked to evaluate the task sensitivity of the scenario (*α* = 0.752). The findings indicated that participants’ reported sensitivity was markedly greater in the high task scenario than in the low task scenario (M _High task sensitivity_ = 3.70, SD = 0.72 vs. M _Low task sensitivity_ = 3.12, SD = 0.75; *t*(83) = 3.627, *p* < 0.001). This validated the effective manipulation of task sensitivity for Study 3.

### 4.4 Experimental procedure

The four formal experiments in this study employed a scenario-based design integrating both text and images, and were based on the methodology utilized by Aaker et al. (2004). Participants were randomly allocated to either the AI or human medical triage doctors condition, and the experimental procedure went as follows: (1) Demographic information (e.g., sex, age, education level) was obtained. (2) Prior attitude assessments (e.g., acceptance of innovative technological products) were conducted. (3) The medical triage consultation scenarios were explained and presented visually. (4) Participants engaged with either the AI or human medical triage interaction screenshots, depending on the condition to which they were assigned (see Supplementary material Appendix 3 for details). (5) The relevant questionnaire assessments were completed. The content and complexity of the task remained consistent across all consultation scenarios. The primary experimental manipulation was the medical triage doctor: either AI or human. To determine the quality and validity of the experimental data, a manipulation check item was incorporated into the questionnaires; specifically, in Studies 1 and 4, after participants viewed the triage interaction screenshots, they were asked the question: “Based on the consultation you have observed, the medical triage doctor is: (1) AI, or (2) human.” In Studies 2 and 3, the question was similar, but the response options were: “(1) low-anthropomorphism AI, (2) high-anthropomorphism AI, or (3) human.” If any participant answered this question incorrectly their data was subsequently excluded from the study.

### 4.5 Sample and data collection

An *a priori* power analysis was conducted using G*Power 3.1 to determine the minimum required sample size for each study. For all analyses, the significance level (*α*) was set at 0.05, statistical power (1-effect) was set at 0.80, and a medium effect size was assumed. Study 1 aimed to examine group differences and a mediation effect. Based on Cohen’s (2013) criteria, a medium effect size was set (*d* = 0.5). Referencing the authoritative sample size recommendations for bootstrap mediation tests by Fritz and MacKinnon (2007), the calculation indicated that a minimum of 128 participants was required. For the subsequent studies, an effect size of *f* = 0.25 was set. Calculations indicated a minimum requirement of 156 participants for Study 2, 210 participants for Study 3, and 128 participants for Study 4.

Participants for this research were recruited online via the Credamo platform data service. Each participant was randomly assigned to one experimental scenario. After removing invalid questionnaires (e.g., those with excessively short completion times or incorrect answers to manipulation check items), a total of 976 valid questionnaires were obtained: Study 1 (*N* = 191), Study 2 (*N* = 210), Study 3 (*N* = 355), and Study 4 (*N* = 220). All final sample sizes met the minimum requirements suggested by the a priori power analysis. The demographic data of the sample are shown in Table 2.

**Table 2:** Demographic data of the four studies sample.

Category	Option	Frequency	Percentage (%)
Sex	Male	483	49.49
	Female	493	50.51
Age	Under 18 years	1	0.10
	18–25	307	31.45
	26–35	375	38.42
	36–45	146	14.96
	46–55	82	8.40
	Over 55	65	6.66
Education level	Middle school or below	62	6.35
	High school/secondary/technical school	96	9.84
	Junior college (associate degree)	117	11.99
	Bachelor’s degree	514	52.66
	Postgraduate (master’s) and above	187	19.16
Occupation	Student	166	17.01
	State-owned enterprise	145	14.86
	Public institution	117	11.99
	Civil servant	77	7.89
	Private enterprise	352	36.07
	Foreign-funded enterprise	102	10.45
	Other	17	1.74

## 5 Data analysis

### 5.1 Study 1: the mediating effect of psychological distance in user trust toward medical triage doctors

We recruited 191 participants (92 males). Ages were concentrated in the ranges of 18–25 years (30.37%) and 26–35 years (51.31%). In the interaction task, participants sought triage assistance for persistent headaches, described as being particularly severe upon waking in the morning (materials available upon request). The psychological distance scale (*α* = 0.828) and the user trust scale (*α* = 0.781) demonstrated good internal consistency.

*Manipulation check*. Participants were required to successfully distinguish between the AI and human medical triage doctors (*p* < 0.001). A one-sample *t*-test against the midpoint (4) of the seven-point scale indicated strong immersion in the scenario (M = 6.225, *t*(190) = 108.564, *p* < 0.001), mitigating concerns that low involvement could bias causal inferences. No between-condition differences were found in receptiveness to novel technologies, trust in AI medical products, or scenario involvement (all *p* > 0.05), ruling out these individual-difference factors as alternative explanations and supporting the effectiveness of the stimuli.

**Figure 2:**
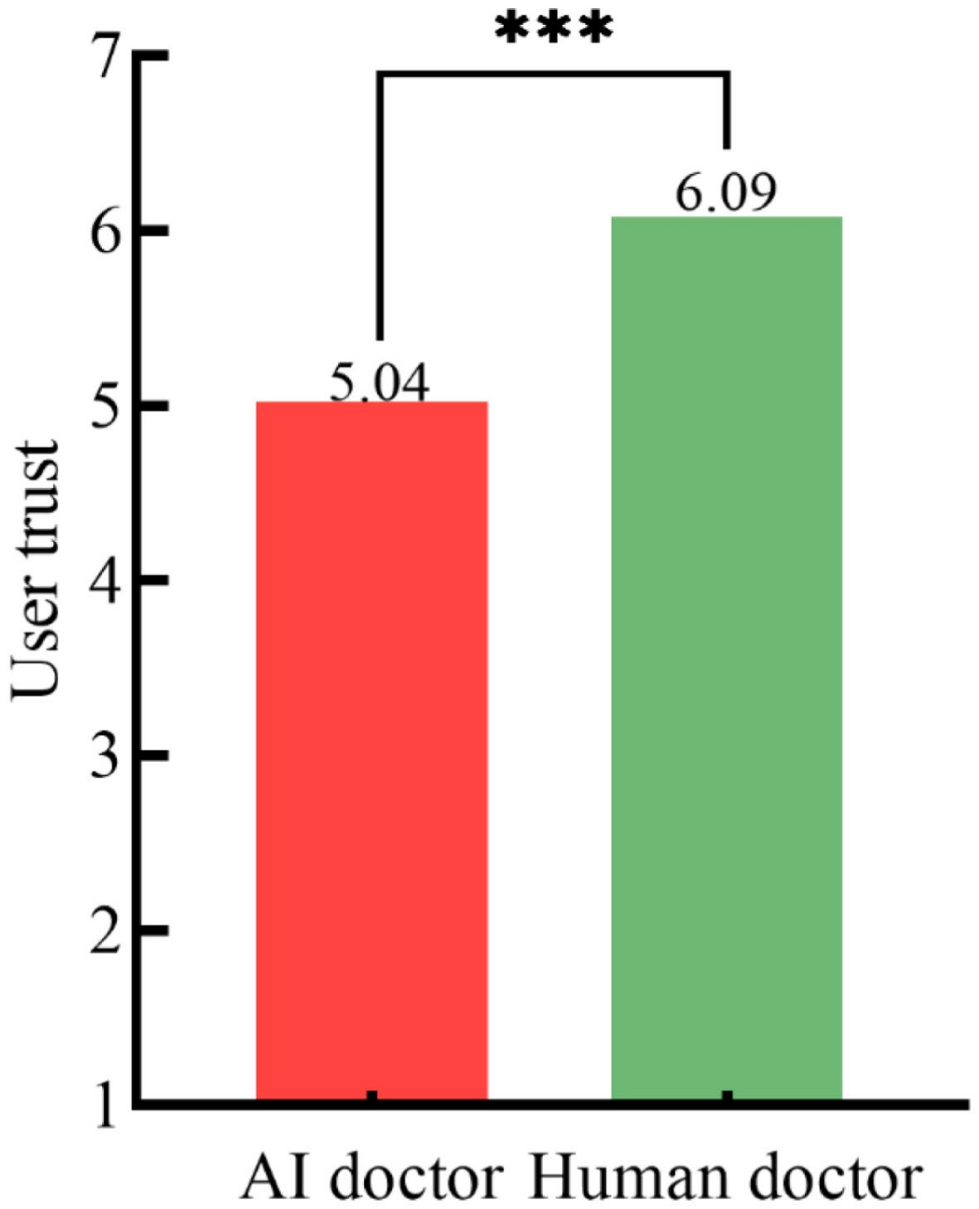
Effect of medical triage doctors on user trust. *** *p* < 0.001; *ns*: *p* > 0.05 (no significant between-group difference).

*Main effect test*. An independent samples *t*-test compared user trust across the medical triage doctors. Trust was significantly higher in the human medical triage doctor condition compared to the AI condition (M_Human medical triage doctor_ = 6.09, *SD* = 0.57 vs. M_AI medical triage doctor_ = 5.04, *SD* = 0.50, *t*(189) = −13.609, *p* < 0.001), supporting H1. The effect of the medical triage doctors on user trust is shown in Figure 2.

A bootstrapping analysis with 5,000 samples was conducted using the PROCESS macro (Hayes, 2013; Model 4) to test the mediating effect of psychological distance on the relationship between the medical triage doctors and user trust. The results (see Table 3) indicated a significant indirect effect, as the 95% confidence interval (CI) for the effect through psychological distance did not include zero (effect = 0.649, 95% BootCI = [0.461, 0.855]). Moreover, the total effect of the medical triage doctor on user trust was significant (effect = 1.056, *t* = 13.609, *p* < 0.001). When controlled for psychological distance, the direct effect of the medical triage doctor on user trust remained significant (effect = 0.407, *t* = 4.947, *p* < 0.001). Since both the direct and indirect effects were significant, the results support a partial mediation model. These findings verify H2.

**Table 3:** Test results of the mediating effect of psychological distance in Study 1 (*N* = 191).

Category	Effect	SE	*t*	*p*	95% CI		Conclusion
					BootLLCI	BootULCI	
Indirect effect	0.649	0.100	-	CI excludes 0	0.461	0.855	Partial mediation
Direct effect	0.407	0.082	4.947	***	0.244	0.569	
Total effect	1.056	0.078	13.609	***	0.903	1.209	

*** *p* < 0.001.

### 5.2 Study 2: the moderating effect of anthropomorphism

We recruited 210 participants (107 males). Ages were concentrated in the ranges of 18–25 years (32.38%) and 26–35 years (45.24%). Apart from adding an anthropomorphism manipulation for the AI medical triage doctor, the procedure and design were identical to Study 1. The anthropomorphism scale (*α* = 0.856) used the pretest items, and the psychological distance (*α* = 0.887) and user trust (*α* = 0.907) measures were the same as in Study 1; all exhibited strong internal consistency.

*Manipulation check*. Participants were required to successfully distinguish between the high- and low- anthropomorphism AI medical triage doctors and human medical triage doctors (*p* < 0.001) and reported experiencing strong immersion in the scenario (*p* < 0.001). No between-condition differences were observed in receptiveness to novel technologies, trust in AI medical products, or scenario involvement (all *p* > 0.05). An independent samples *t*-test confirmed that perceived anthropomorphism was higher in the high-anthropomorphism medical triage doctor condition than in the low-anthropomorphism AI condition (M _High-anthropomorphism AI medical triage doctor_ = 5.60, *SD* = 0.60 vs. M _Low-anthropomorphism AI medical triage doctor_ = 4.14, SD = 0.59; *t*(138.000) = −14.442, *p* < 0.00). These results indicated that the anthropomorphism manipulation was successful, as the participants clearly differentiated between the high–low-anthropomorphism AI medical triage doctors; thus, the stimuli were effective.

*Main effect test*. An independent samples *t*-test showed that user trust was higher in the human medical triage doctor condition than in the AI condition (M_Human medical triage doctor_ = 6.02, SD = 0.63 vs. M_AI medical triage doctor_ = 4.80, SD = 1.04; *t*(199.800) = −10.577, *p* < 0.001), thereby again confirming H1.

*Mediation analysis*. The mediation analysis was repeated using the PROCESS macro (Model 4). Results showed that the total effect of the medical triage doctors on user trust was significant (effect = 1.223, *t* = 9.066, *p* < 0.001), and the direct effect also remained significant (effect = 0.457, *t* = 5.217, *p* < 0.001). Furthermore, the bootstrap analysis confirmed a significant indirect effect through psychological distance (effect = 0.767, 95% BootCI = [0.557, 0.997]). As detailed in Table 4, these findings once again supported the partial mediating role of psychological distance, further verifying H2.

**Table 4:** Test results of the mediating effect of psychological distance in Study 2 (*N* = 210).

Category	Effect	SE	*t*	*p*	95% CI		Conclusion
					BootLLCI	BootULCI	
Indirect effect	0.767	0.112	-	CI excludes 0	0.557	0.997	Partial mediation
Direct effect	0.457	0.088	5.217	***	0.284	0.629	
Total effect	1.223	0.135	9.066	***	0.957	1.489	

*** *p* < 0.001.

**Figure 3:**
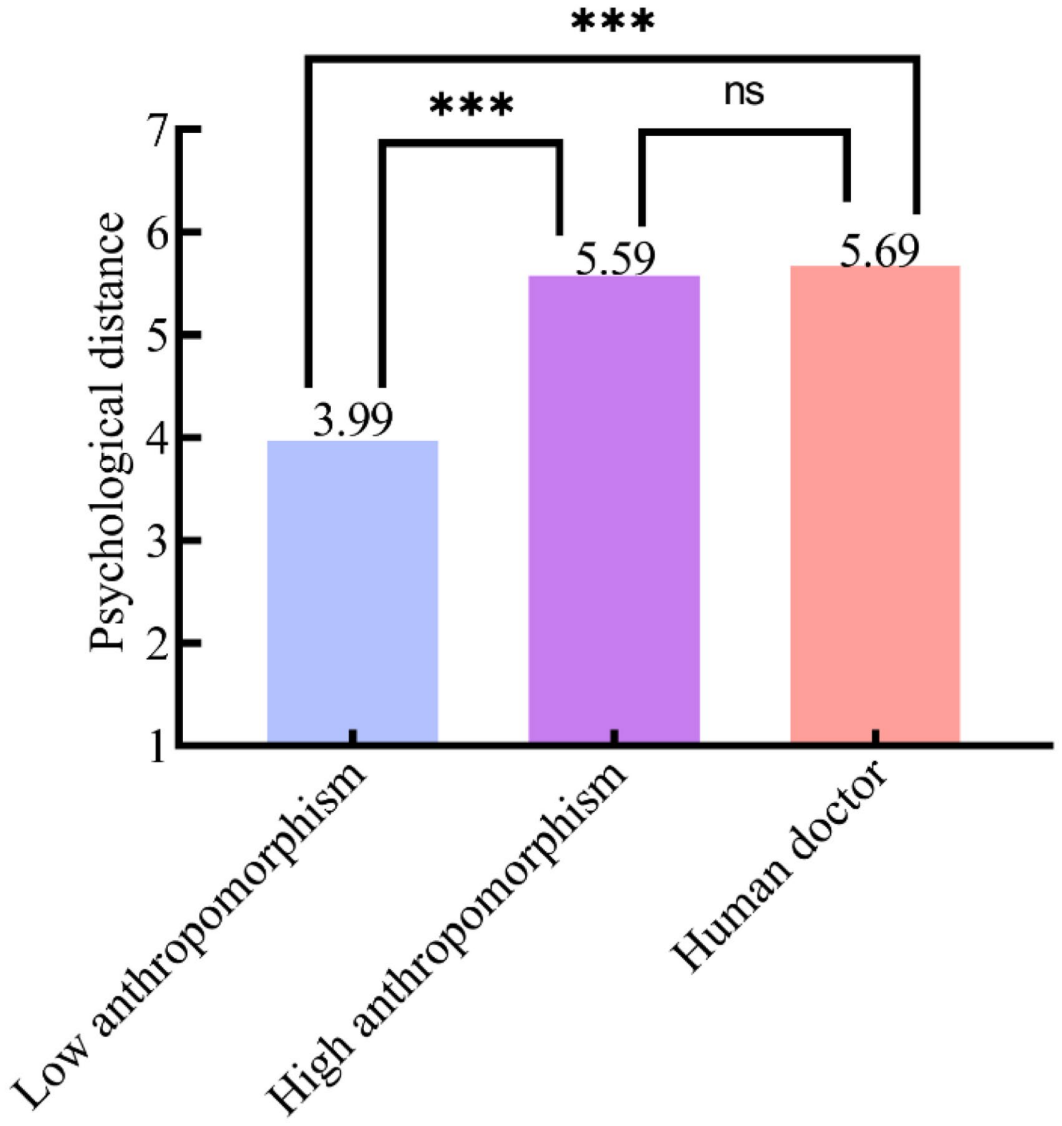
Interaction effect of medical triage doctors and anthropomorphism on psychological distance. *** *p* < 0.001; ns: *p* 0.05; no significant between-group difference.

Moderation analysis. Because anthropomorphism was manipulated only for the AI medical triage doctor, three one-way ANOVAs and *post hoc* multiple-comparison tests were conducted to examine whether anthropomorphism moderated the effect of the medical triage doctor on psychological distance. Results (see Table 5) showed a significant main effect of the medical triage doctor [*F*(1, 208) = 48.849, *p* < 0.001], a significant main effect of anthropomorphism [*F*(1, 138) = 216.908, *p* < 0.001], and a significant effect for the three-level factor [low-anthropomorphism AI vs. high-anthropomorphism AI vs. human medical triage doctors; *F*(1, 207) = 148.397, *p* < 0.001]. *Post hoc* tests (see Table 6) indicated that, relative to those in the low-anthropomorphism AI medical triage doctor condition, participants in the human medical triage doctor condition reported smaller psychological distance (M_Human medical triage doctor_ = 5.69, SD = 0.72 vs. M_Low-anthropomorphism AI medical triage doctor_ = 3.99, SD = 0.66, *p* < 0.001), supporting H3a. Reported psychological distance in the high-anthropomorphism AI medical triage doctor condition did not differ from that in the human medical triage doctor condition (M_High-anthropomorphism AI medical triage doctor_ = 5.59, SD = 0.63 vs. M_Human medical triage doctor_ = 5.69, SD = 0.72, *p* = 0.398 > 0.05), supporting H3b. In sum, anthropomorphism moderated the effect of the medical triage doctor on psychological distance; H3, H3a, and H3b were all supported. The mean plot for the medical triage doctor × anthropomorphism comparison is shown in Figure 3.

**Table 5:** ANOVA results of psychological distance for variables tested in Study 2 from medical triage doctors.

Variable	Medical triage doctors (M ± SD)		*F*	*p*
	AI medical triage doctor (*n* = 140)	Human medical triage doctor (*n* = 70)		
Psychological distance	4.73 ± 1.03	5.69 ± 0.72	48.849	***

Variable	Anthropomorphism (M ± SD)		*F*	*p*
	Low-anthropomorphism AI medical triage doctor (*n* = 75)	High-anthropomorphism AI medical triage doctor (*n* = 65)		
Psychological distance	3.99 ± 0.66	5.59 ± 0.63	216.908	***

Variable	Different medical triage doctors (M ± SD)			*F*	*p*
	Low-anthropomorphism AI medical triage doctor (*n* = 75)	High-anthropomorphism AI medical triage doctor (*n* = 65)	Human medical triage doctor (*n* = 70)		
Psychological distance	3.99 ± 0.66	5.59 ± 0.63	5.69 ± 0.72	148.397	***

*** *p* < 0.001.

**Table 6:** Post hoc multiple comparison results in Study 2.

Category	(I) Group	(J) Group	(I) M	(J) M	(I−J)	SE	*p*
Psychological distance	Low-anthropomorphism AI medical triage doctor	High-anthropomorphism AI medical triage doctor	3.99	5.59	−1.60	0.113	***
	Low-anthropomorphism AI medical triage doctor	Human medical triage doctor	3.99	5.69	−1.70	0.111	***
	High-anthropomorphism AI medical triage doctor	Human medical triage doctor	5.59	5.69	−0.10	0.115	0.398

(I) Name and (J) Name indicate the two groups being compared in each post hoc pairwise comparison. I is the reference group, and J is the comparison group. (I) Mean and (J) Mean are the group means for groups I and J, respectively. Difference (I−J) is the mean difference, calculated as (I) Mean−(J) Mean. A negative (I−J) value means the mean of group I is lower than the mean of group J; a positive (I−J) value means the mean of group I is higher than that of group J and *** *p* < 0.001.

### 5.3 Study 3: the higher-order moderating effect of task sensitivity

We recruited 355 participants (172 males). Ages were concentrated in the ranges of 18–25 years (27.32%) and 26–35 years (30.42%). Aside from adding manipulations of task sensitivity and anthropomorphism for the AI medical triage doctor, as well as varying the interaction script by task sensitivity, the procedure matched that of Study 1 and Study 2. The anthropomorphism scale (*α* = 0.905) and task sensitivity scale (*α* = 0.860) adopted the pretest items, and the measures of psychological distance (*α* = 0.882) and user trust (*α* = 0.938) were the same as in Study 1; all showed strong internal consistency.

*Manipulation check*. Participants were required to successfully distinguished between high- and low-anthropomorphism AI medical triage doctors and human medical triage doctor (*p* < 0.001) and reported strong immersion in the scenario (*p* < 0.001). No between-group differences were observed in receptiveness to novel technologies, trust in AI medical products, or scenario involvement (all *p* > 0.05), indicating that the stimuli were effective. Independent samples *t*-testing confirmed that perceived anthropomorphism was higher in the high-anthropomorphism AI medical triage doctor condition than in the low-anthropomorphism AI condition (M_High-anthropomorphism AI medical triage doctor_ = 5.66, SD = 0.62 vs. M_Low-anthropomorphism AI medical triage doctor_ = 3.09, SD = 0.64; *t*(353.000) = −38.059, *p* < 0.05), and that perceived task sensitivity was higher in the high sensitivity group than in the low sensitivity group (M_High task sensitivity_ = 4.87, SD = 0.83 vs. M_Low task sensitivity_ = 2.98, SD = 0.94; *t*(353.000) = −19.188, *p* < 0.001). These results indicated that both the anthropomorphism and task sensitivity manipulations were successful.

*Moderation analysis*. As Table 7 shows, ANOVA revealed a significant main effect of medical triage doctors [low-anthropomorphism AI medical triage doctor vs. high-anthropomorphism AI vs. human medical triage doctor; *F*(2, 349) = 378.690, *p* < 0.001]; a significant main effect of task sensitivity [*F*(1, 349) = 23.604, *p* < 0.001]; and a significant interaction effect of medical triage doctor × task sensitivity interaction [*F*(2, 349) = 48.211, *p* < 0.001]. Simple-effects tests showed that under the low task sensitivity condition, psychological distance in the high-anthropomorphism AI medical triage doctor condition did not differ from that in the human medical triage doctor condition (M_High-anthropomorphism AI medical triage doctor_ = 5.38, SD = 0.56 vs. M_Human medical triage doctor_ = 5.34, SD = 0.57; *p* = 1.000 > 0.05). Participants in the low-anthropomorphism AI medical triage doctor condition reported significantly greater psychological distance than those in both the high-anthropomorphism AI medical triage doctor condition (M_Low-anthropomorphism AI medical triage doctor_ = 3.36, SD = 0.86; *p* < 0.001) and the human medical triage doctor condition (*p* < 0.001), supporting H4a. Under high task sensitivity, participants in the human medical triage doctor condition reported significantly smaller distance than those in the high-anthropomorphism AI medical triage doctor condition (M_Human medical triage doctor_ = 5.64, SD = 0.56; M_High-anthropomorphism AI medical triage doctor_ = 3.98, SD = 0.84; *p* < 0.001), and those in the high-anthropomorphism AI medical triage doctor condition reported smaller distance than those in the low-anthropomorphism AI condition (M_Low-anthropomorphism AI medical triage doctor_ = 2.91, SD = 0.53; *p* < 0.001), supporting H4b.

**Table 7:** Interaction effects of different medical triage doctors and task sensitivity in Study 3.

Source	Sum of squares	*df*	Mean square	*F*	*p*
Intercept	6977.799	1	6977.799	15616.401	***
Low-anthropomorphism AI medical triage doctor vs. high-anthropomorphism AI medical triage doctor vs. human medical triage doctor	338.417	2	169.208	378.690	***
Task sensitivity	23.604	1	23.604	52.826	***
Different medical triage doctors × task sensitivity	43.084	2	21.542	48.211	***
Residual	155.942	349	0.447		

*R*^2^ = 0.541, *** *p* < 0.001.

**Figure 4:**
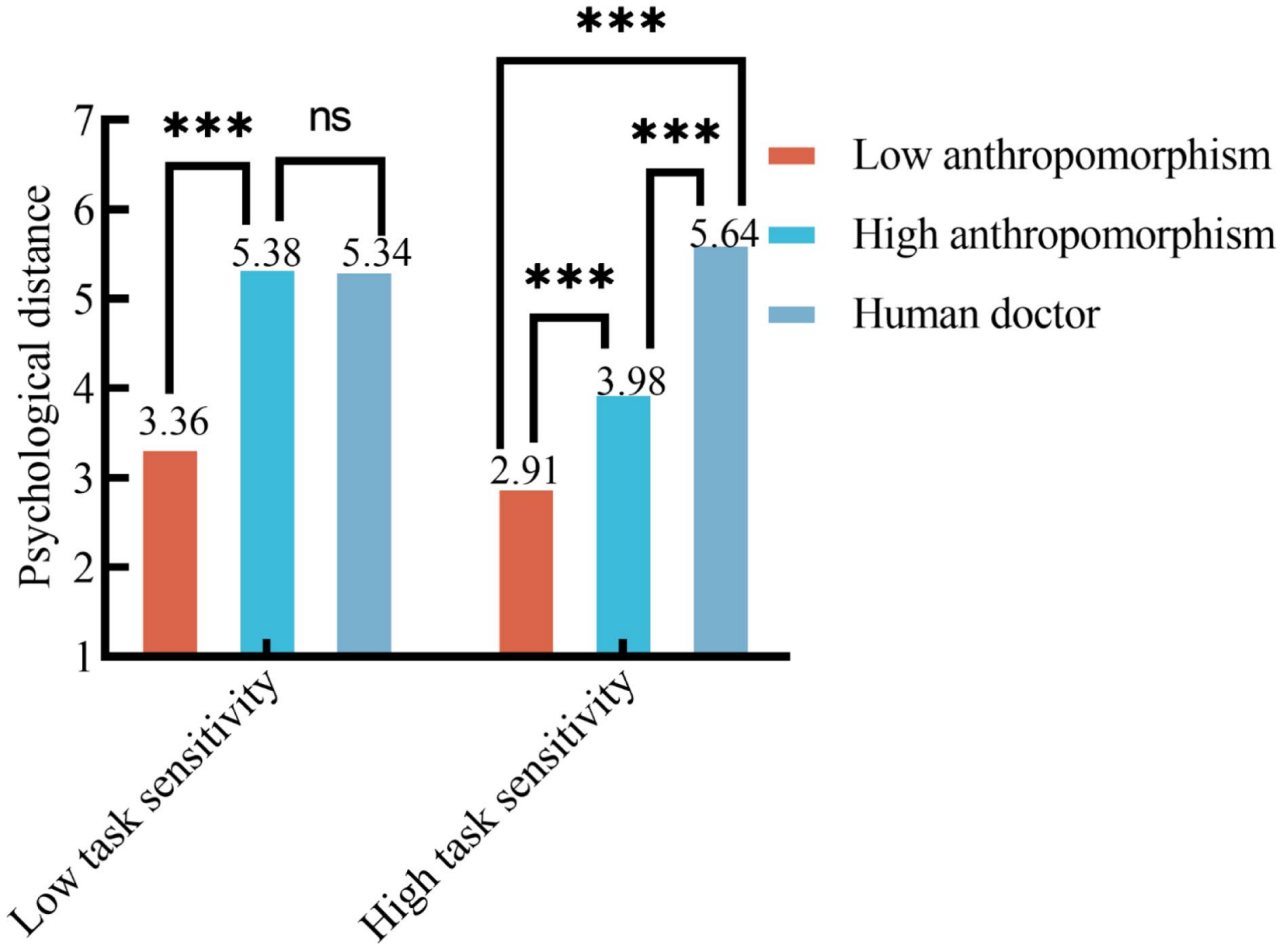
Interaction effect of medical triage doctors, anthropomorphism, and task sensitivity on psychological distance. *** *p* < 0.001.

Taken together, anthropomorphism moderated the effect of the medical triage doctor on psychological distance, and this moderating effect was further conditioned by task sensitivity; thus H4, H4a, and H4b were all supported. The mean comparison for the medical triage doctor × task sensitivity interaction is displayed in Figure 4.

### 5.4 Study 4: the moderating effect of level of AI technology adoption

We recruited 220 participants (112 males). Ages were concentrated in the ranges of 18–25 years (38.18%) and 26–35 years (33.64%). The procedure matched that of Study 1, except for the addition of measuring participants’ AI technology adoption level at the outset (*α* = 0.903), and splitting participants into high vs. low AI technology adoption groups using the sample mean. Measures of psychological distance (*α* = 0.928) and user trust (*α* = 0.915) were identical to those in Study 1.

*Manipulation check*. Participants were required to successfully distinguish between the AI and human medical triage doctors (*p* < 0.001) and reported strong immersion in the scenario (*p* < 0.001). No between-group differences were observed in receptiveness to novel technologies, trust in AI medical products, or scenario involvement (all *p* > 0.05), indicating that the stimuli were effective. An independent samples *t*-test showed that perceived AI technology adoption level was higher in the high AI technology adoption level group than in the low AI technology adoption level group (M_High AI technology adoption level_ = 5.98, SD = 0.46 vs. M_Low AI technology adoption level_ = 3.61, SD = 0.86; *t*(152.753) = −24.950, *p* < 0.001), confirming that the AI technology adoption level grouping was successful.

*Main effect test*. An independent-samples *t*-test showed that user trust was higher for participants in the human medical triage doctor condition than it was for those in the AI condition (M_Human medical triage doctor_ = 5.49, SD = 1.00 vs. M_AI medical triage doctor_ = 4.62, SD = 1.41; *t*(185.535) = −5.262, *p* < 0.001), thus confirming H1 once again.

*Mediation analysis*. The mediation analysis was repeated using the PROCESS macro (Model 4). Results indicated a significant total effect of the medical triage doctor on user trust (effect = 0.877, *t* = 5.342, *p* < 0.001) as well as a significant direct effect (effect = 0.450, *t* = 3.672, *p* < 0.001). A bootstrap analysis also confirmed a significant indirect effect of the medical triage doctor on user trust through psychological distance (effect = 0.427, 95% BootCI = [0.203 to 0.681]), as detailed in Table 8. These results support the partial mediating role of psychological distance, further verifying H2.

**Table 8:** Test of the mediating effect of psychological distance in Study 4 (*N* = 220).

Category	Effect	SE	*t*	*p*	95% CI		Conclusion
					BootLLCI	BootULCI	
Indirect effect	0.427	0.122	-	CI excludes 0	0.203	0.681	Partial mediation
Direct effect	0.450	0.123	3.672	***	0.208	0.691	
Total effect	0.877	0.164	5.342	***	0.553	1.200	

*** *p* < 0.001.

**Figure 5:**
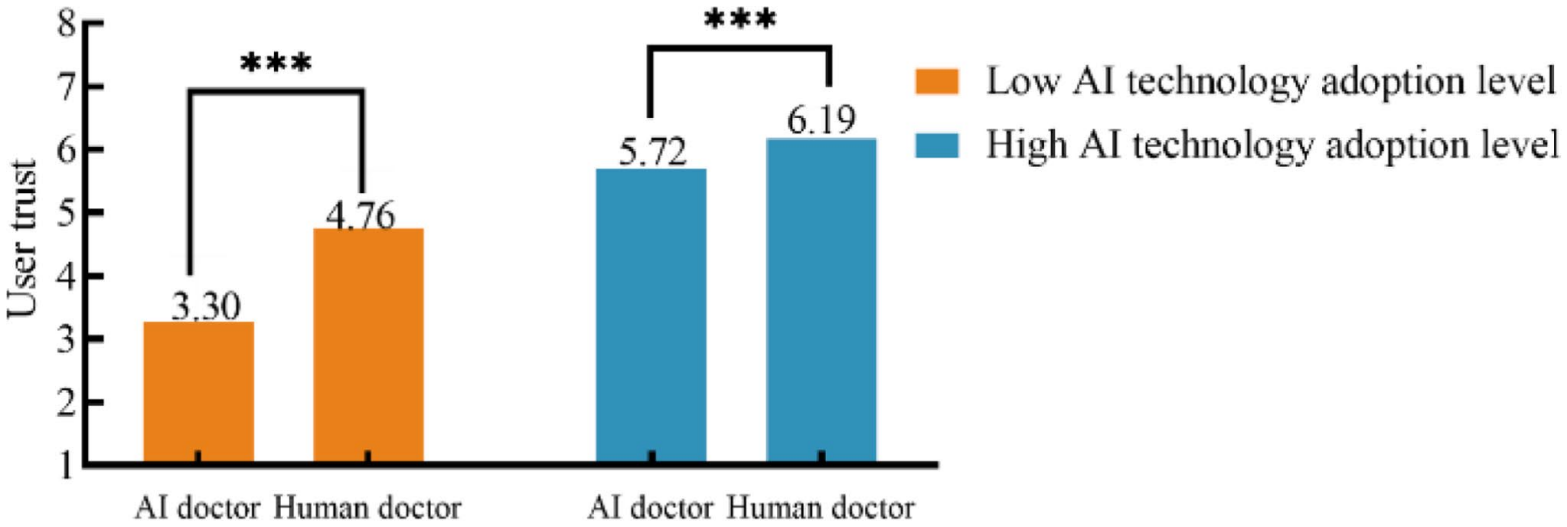
Interaction effect of medical triage doctors and AI technology adoption level on user trust.

*Moderation analysis*. As Table 9 shows, ANOVA revealed a significant main effect of medical triage doctor [*F*(1, 216) = 98.143, *p* < 0.001], a significant main effect of AI technology adoption level [*F*(1, 216) = 391.863, *p* < 0.001], and a significant interaction effect of medical triage doctor × AI technology adoption level interaction [*F*(1, 216) = 26.434, *p* < 0.001]. Specifically, among participants with a high level of AI technology adoption, user trust was higher in the human medical triage doctor condition than it was in the AI condition (M_AI medical triage doctor_ = 5.72, SD = 0.34 vs. M_Human medical triage doctor_ = 6.19, SD = 0.46, *F*(1, 216) = 107.081, *p < 0*.001); thus, H5a was not supported. Among participants with a low level of AI technology adoption, user trust was higher in the human medical triage doctor condition than it was in the AI condition (M_AI medical triage doctor_ = 3.30, SD = 1.02 vs. M_Human medical triage doctor_ = 4.76, SD = 0.89, *F*(1, 216) = 12.048, *p* < 0.001), supporting H5b. In sum, a higher level of AI technology adoption increased trust in the AI medical triage doctor and narrowed the trust gap with the human medical triage doctor; overall, H5 and H5b were supported, whereas H5a was not. The mean comparison for the medical triage doctor × AI technology adoption level interaction is shown in Figure 5.

**Table 9:** Interaction effects of medical triage doctors and level of AI technology adoption in Study 4.

Source	Sum of squares	*df*	Mean square	*F*	*p*
Intercept	5452.386	1	5452.386	10578.416	***
Medical triage doctors	50.586	1	50.586	98.143	***
AI technology adoption level	201.976	1	201.976	391.863	***
Medical triage doctors × AI technology adoption level	13.625	1	13.625	26.434	***
Residual	111.332	216	0.515		

*R*^2^ = 0.695, *** *p* < 0.001.

## 6 Conclusion

After systematically reviewing the literature on AI healthcare and medical triage, this study identified a mediated pathway by which the medical triage doctor (both AI and human) affects user trust through psychological distance, and empirically evaluated this mechanism using multiple moderators. The core findings are as follows:

First, medical triage doctors (both AI and human) significantly affected user trust, with psychological distance serving as a partial mediator. Relative to the AI medical triage doctors, the human medical triage doctor decreased psychological distance, thereby increasing user trust. Beyond this indirect pathway, the medical triage doctor also had a direct effect, in that the human medical triage doctor elicited higher user trust than an AI medical triage doctor.

Second, higher anthropomorphism shortened psychological distance and increased trust in AI medical triage doctors. When the AI medical triage doctor was perceived as being high in anthropomorphism, participants reported less psychological distance and greater trust. Elevated anthropomorphism thereby mitigates part of the AI medical triage doctor’s disadvantage relative to human medical triage doctors, yielding trust levels comparable to human triage.

Third, the effect of anthropomorphism was constrained by context and further influenced by task sensitivity. The moderating effect of anthropomorphism was contingent upon the situation and additionally influenced by task sensitivity. In low task sensitivity scenarios, a high-anthropomorphism AI medical triage doctor diminished psychological distance to a point comparable to that of a human doctor, with both surpassing a low-anthropomorphism AI medical triage doctor. This indicates that for routine, low sensitivity consultations, high-anthropomorphism AI medical triage doctors can effectively replace a human medical triage doctor. Conversely, in high task sensitivity scenarios, the irreplaceability of human medical triage doctors becomes evident: participants experienced the closest psychological distance – and consequently the greatest trust – with the human medical triage doctor, followed by the high-anthropomorphism AI medical triage doctor, and finally the low-anthropomorphism AI medical triage doctor. In situations characterized by significant privacy concerns or severity, increased anthropomorphism is beneficial, however, users maintain the strongest affinity for and greatest faith in human medical triage doctors in these circumstances.

Fourth, AI technology adoption level moderated group differences, but the human medical triage doctor’s advantage persisted. AI technology adoption level was found to moderate the effect of the medical triage doctors (both AI and human) on user trust. Users with a low level of AI technology adoption showed greater trust in the human medical triage doctor group than in the AI medical triage doctor condition. Among users with a high level of AI technology adoption, acceptance of AI increased participants’ trust in the AI medical triage doctor and narrowed the trust gap with the human medical triage doctor, however, a preference for human medical triage doctors nonetheless remained. In short, the trust advantage of the human medical triage doctor was maintained consistently across all groups. This further suggests that, in the context of medical triage, a general preference for AI technology is not sufficient to fully offset users’ need for psychological security, nor their concerns about algorithmic accountability and system safety. Even among high AI adopters, trust in AI medical triage doctors may be undermined by the perceived ambiguity of responsibility when clinical decision errors occur, as well as by perceived uncontrollable risks associated with unexpected technical malfunctions. In contrast, human medical triage doctors possess legal and ethical moral agency and can be held clearly accountable for medical outcomes. This perceived traceability of responsibility functions as an indispensable psychological safety safeguard. Moreover, users may view uniquely human capacities – such as emotional communication, reassurance, a sense of moral obligation, and the ability to manage sudden, non-routine, or unstructured complex problems – as core advantages that AI medical triage doctors cannot yet fully replicate.

## 7 Implications and future directions

### 7.1 Theoretical contributions

First, this study brings psychological distance into the exploration of the AI medical triage context and clarifies its mediating effect in the formation of user trust. Prior research has placed great emphasis on the Technology Acceptance Model and anthropomorphism, with comparatively little attention given to users’ deeper psychological perceptions. By foregrounding psychological distance as a core mediator, we systematically traced how trust emerges across different types of medical triage doctors (AI vs. human) and provide a complementary framework for understanding trust formation in AI medical triage doctors.

Second, this research unpacks the context dependence of AI anthropomorphism in medical triage. Although many studies have found that anthropomorphism design can reduce psychological distance and thereby increase user trust, our introduction of task sensitivity as a second-stage moderator demonstrates that these benefits are not universal, but rather contingent on the clinical context. Under low sensitivity task conditions, high-anthropomorphism AI medical triage doctors are able to meaningfully shorten users’ perceived psychological distance and achieve trust levels comparable to human medical triage doctors; under high sensitivity task conditions, the trust gains from anthropomorphism are limited, and users continue to place greater trust in human medical triage doctors. This finding offers actionable guidance for differentiated design of AI medical triage services; specifically, the applicability of anthropomorphism should be evaluated carefully across tasks with different risk and privacy profiles.

Third, this study advances a comprehensive trust model that integrates multiple moderators within a single framework. Moving beyond single-variable accounts, we have incorporated psychological distance, anthropomorphism, task sensitivity, and level of AI technology adoption into a unified model and examined their interplay. The model not only corroborates the core pathways of trust formation in AI medical triage doctors but also reveals the complexity and dynamics of the trust-building process.

### 7.2 Practical implications

The findings of this research offer meaningful managerial implications for guiding healthy development of smart AI healthcare.

First, implement differentiated, context-aware AI deployment. When introducing AI medical triage doctors, organizations should avoid positioning them as one-to-one substitutes for human care and instead adopt human–AI collaborative deployment. For low sensitivity tasks (e.g., appointment scheduling, department navigation), high-anthropomorphism, high-capability AI can be deployed to improve efficiency. For high sensitivity tasks (e.g., privacy-laden issues or serious-condition consultations), organizations should default to using human or AI + human oversight models, with guaranteed human back-up available for when circumstances require it. If AI must be used, authoritative endorsement, transparent risk disclosures, and seamless escalation/hand-off mechanisms should be provided to mitigate potential losses in trust.

Second, incorporate psychological distance as a core design parameter for AI healthcare services. Advancing intelligent transformation requires attention not only to technical functionality and efficiency, but also to the perceived psychological proximity between the technology and its users. AI medical triage doctors should employ inclusive, empathetic, and human-centered design, language, and patterns of interaction. Humanizing cues such as compassionate responses, clear explanations, and timely apologies can narrow the psychological gap between users and AI services, thereby strengthening user trust. Elevating psychological distance to a fundamental design consideration is critical for sustaining the perceived legitimacy and acceptability of AI healthcare services.

Third, establish a trust-centric AI governance and evaluation framework. In assessing AI healthcare services, user trust should be a core dimension, alongside technical performance. Strategies should be tailored to user segments: for individuals with low AI adoption, prioritize an AI + human backup model that preserves clinician oversight to enhance perceived safety and controllability; for users with high AI acceptance, increase AI autonomy while strengthening explainability and transparency to meet high expectations and reinforce user confidence.

### 7.3 Limitations and future research

This study has several limitations. First, the sample lacks broad representativeness, being concentrated in specific online and student populations that differ from the general public in age, education, and digital literacy; therefore, the findings may not generalize well to wider groups (e.g., older adults, rural residents). Second, there are inherent limitations regarding the experimental manipulation and the ecological validity of the scenario simulations, meaning that the scenario-based experiment is simplified relative to real clinical encounters. Specifically, for the manipulation of anthropomorphism, the high-anthropomorphism AI medical triage doctor was presented using an image of a human doctor while explicitly labeled as an AI doctor. Although the manipulation-check results indicated that participants understood and accepted the doctor as a high-anthropomorphism AI medical triage doctor, this combination of visual appearance and identity labeling may still have introduced perceptions of identity ambiguity or potential deception cues. In addition, although the text- and image-based design aids can be used in causal inference, they cannot fully reproduce the complex emotions and behavioral decisions present in real interactions, where further additional uncontrolled factors may also be operating in practice. Third, the measurement approach used in these studies was relatively single-method: core constructs relying on self-report scales, with limited validation from physiological or behavioral data.

To address these limitations, future work should proceed along three directions. (1) Broaden sampling coverage: use stratified sampling across multiple regions and recruitment channels, explicitly including older adults, rural residents, and individuals with lower digital literacy to enhance external validity. Future studies could also conduct cross-age or cross-cultural comparisons to examine whether the trust mechanisms underlying reliance on AI medical triage doctors differs across populations, thereby providing stronger evidence for the construction of more generalizable smart healthcare services. (2) The experimental manipulation should be refined, and the scenario realism improved. To minimize potential confounds in the manipulation of anthropomorphism (e.g., identity ambiguity and deception cues), future research could use high-fidelity computer-generated imagery (CGI) or GAN-based methods to create ultra-realistic “digital human” medical triage doctors, allowing for a cleaner test of the effects of anthropomorphism appearance. In addition, researchers should move from static text-and-image vignettes toward more dynamic, interactive paradigms – such as high-simulation chatbots or immersive VR-based clinical environments – to better approximate actual doctor–patient dialogue, emotional exchange, and real-time feedback. Such designs can also be paired with longitudinal (repeated-measures) approaches to trace the dynamic evolution of trust, psychological distance, and affective bonding over sustained use of AI medical triage doctors. (3) Multimethod measurement and triangulation should be adopted. Self-report data should be complemented by physiological indices (e.g., heart rate variability, electrodermal activity) and objective behavioral data (e.g., eye-tracking trajectories, fixation duration, decision response time, interaction frequency and duration, and actual adherence/behavioral compliance) to build a subjective–objective validation framework and capture deeper psychological processes during human and AI medical triage doctors more precisely.

## Data Availability

The original contributions presented in the study are included in the article/Supplementary material, further inquiries can be directed to the corresponding author.
