# Supplementary material for: Who is more trustworthy? The influence mechanism of AI vs. human doctor triage on user trust: testing the mediating effect of psychological distance, and a multiple moderating effects analysis
Source: Front Psychol. 2026 Jan 12;16:1730902. doi: 10.3389/fpsyg.2025.1730902 (PMC12832618; doi:10.3389/fpsyg.2025.1730902)
Supplement: Supplementary file 1 [file Table_1.DOCX]

Supplementary Material

# Supplementary Figures

# Appendix 1


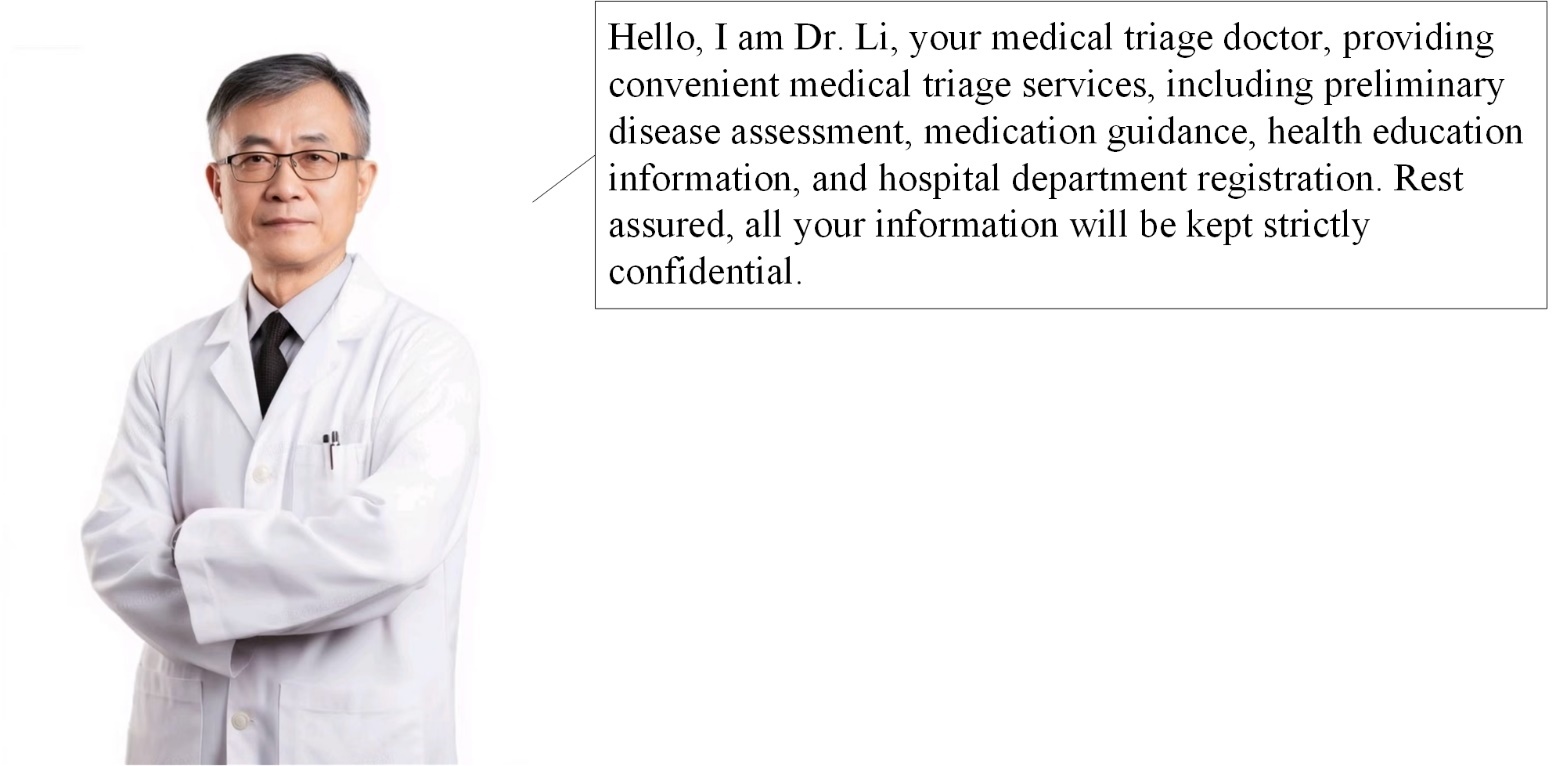


Initial interface of the human doctor


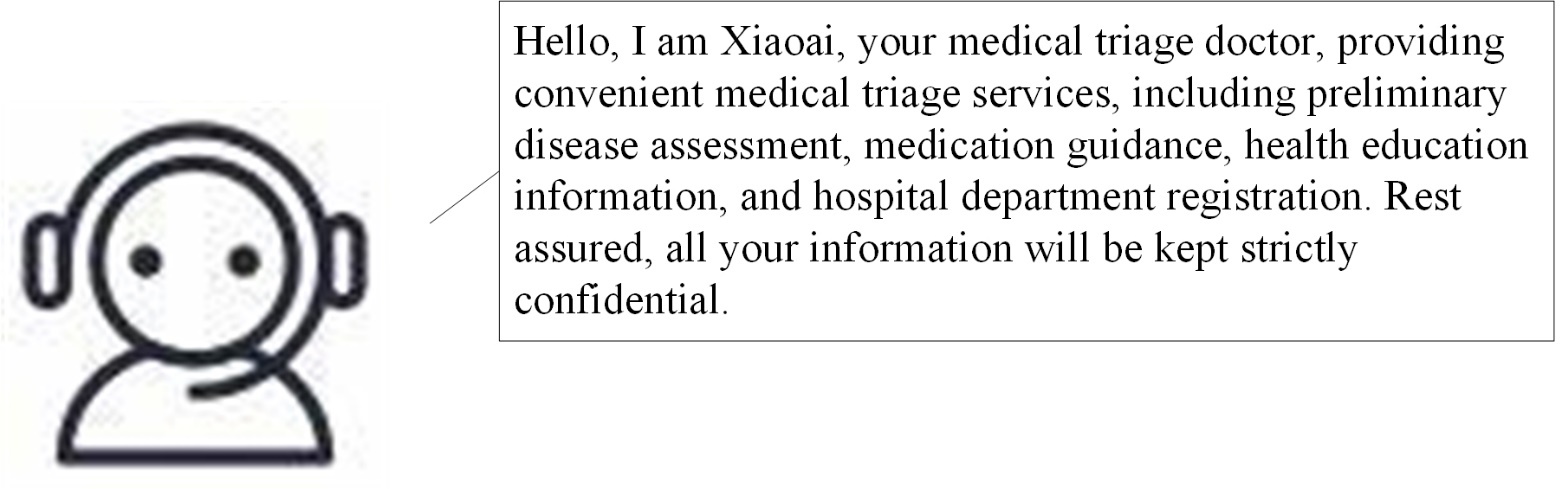


Initial interface of the AI doctor

# Appendix 2


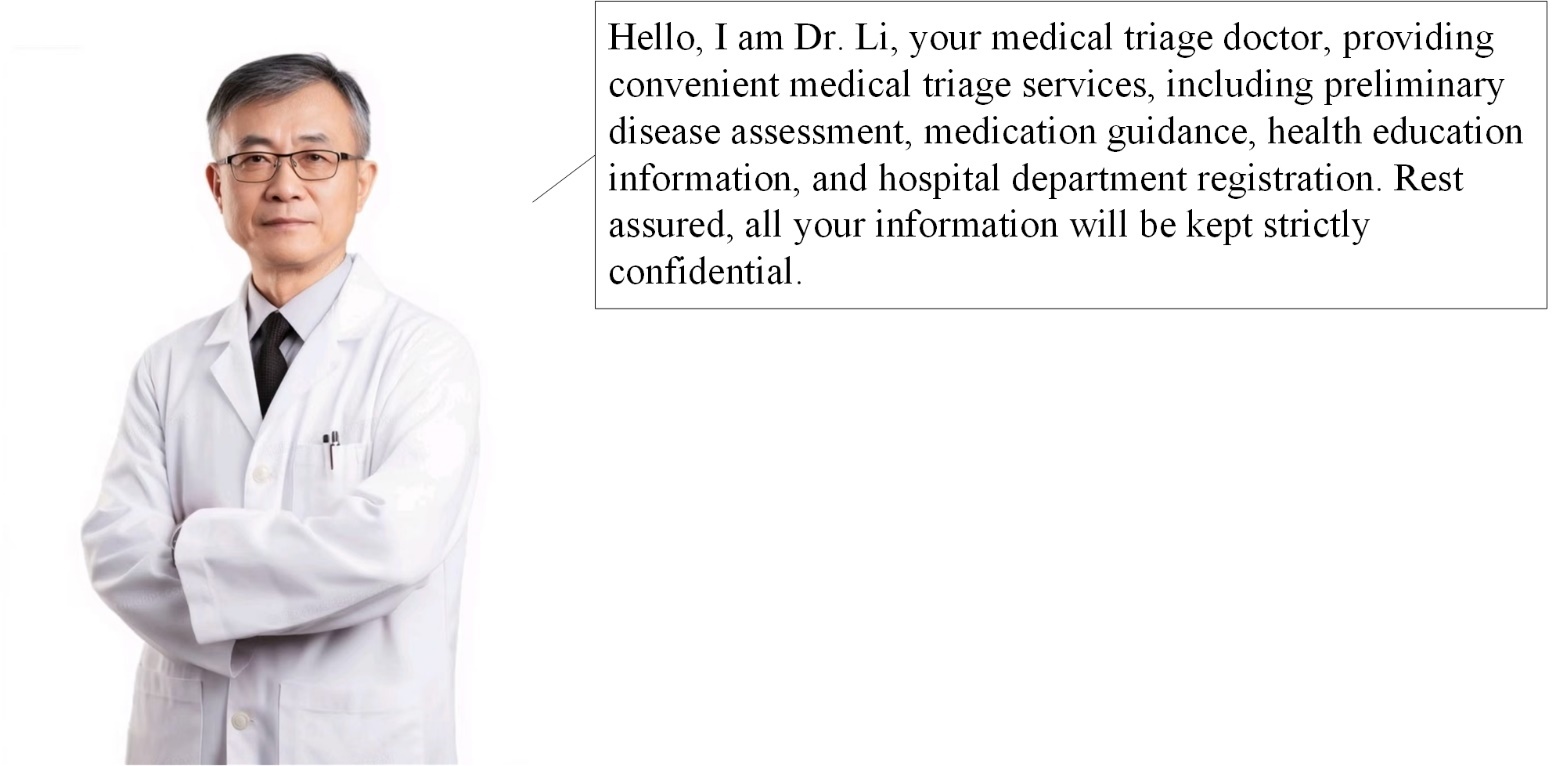


Initial interface of the human doctor


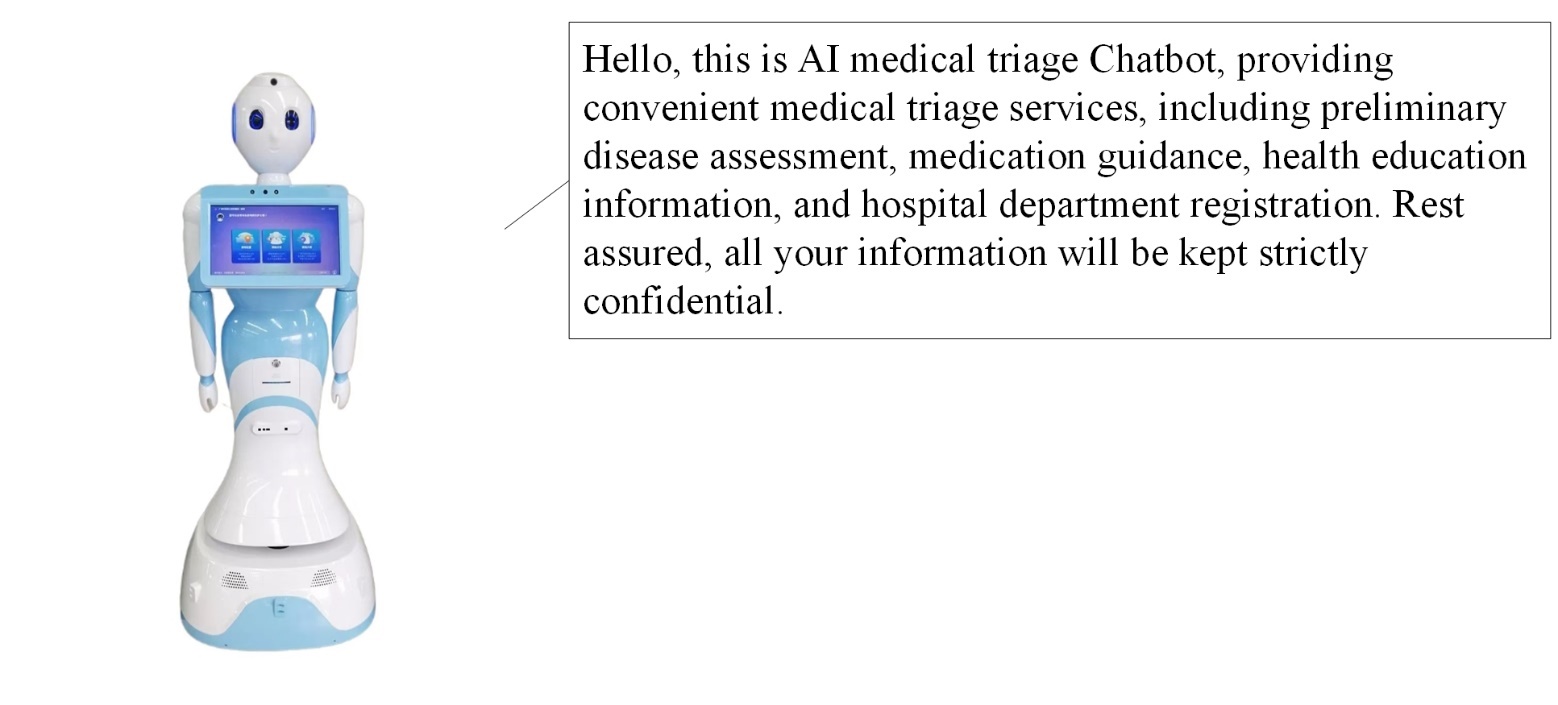


Initial interface of the low anthropomorphism AI doctor


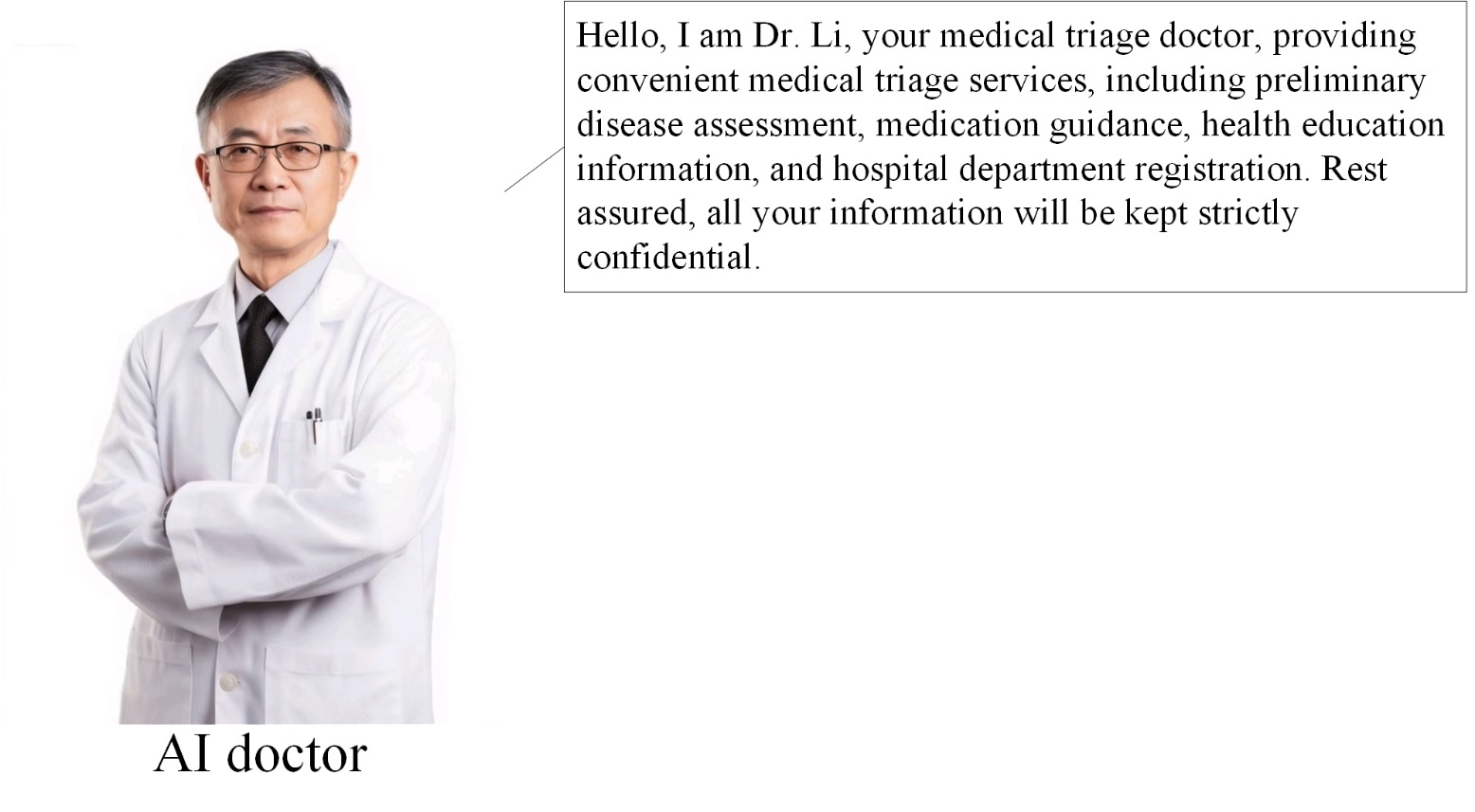


Initial interface of the high anthropomorphism AI doctor

# Appendix 3

Study 1, Study 2, and Study 4: Human doctor group interaction content

Study 1 and Study 4: AI doctor group interaction content

Study 2: Low anthropomorphism AI doctor group interaction content

Study 2: High anthropomorphism AI doctor group interaction content

Study 3: Low task sensitivity human doctor group interaction content

Study 3: Low anthropomorphism、low task sensitivity AI doctor group interaction content

Study 3: High anthropomorphism、 low task sensitivity AI-doctor group interaction content

Study 3: High task sensitivity human doctor group interaction content

Study 3: High anthropomorphism 、high task sensitivity AI-doctor group interaction content

Study 3: Low anthropomorphism 、high task sensitivity AI-doctor group interaction conten
